# Supplementary material for: Measuring health financing vulnerability due to reductions in official development assistance: A conceptual framework with empirical application across 47 African countries
Source: PLOS Glob Public Health. 2026 May 6;6(5):e0006282. doi: 10.1371/journal.pgph.0006282 (PMC13148685; doi:10.1371/journal.pgph.0006282)
Supplement: S1 File — (DOCX) [file pgph.0006282.s002.docx]

**S1 File: Vulnerability Assessment by Country**

| **Country** | **CHE Per Capita (2022)** | **Foreign Aid Dependency** | **Budget Space Potential** | **Overall Risk of Debt Distress (Capacity to Borrow)** |
| --- | --- | --- | --- | --- |
| Algeria | 60th percentile - with US$179.7 CHE per capita in 2022 | Very Low Dependency - 0.5% | Contraction | N/A |
| Angola | 60th percentile - with US$101.3 CHE per capita in 2022 | Very Low Dependency - 4.7% | Contraction | N/A |
| Benin | 20th percentile (lower quintile) - with US$33.9 CHE per capita in 2022 | High Dependency. - 32% | Expansion | Moderate |
| Botswana | 80th percentile (Upper quintile) - with US$477.8 CHE per capita in 2022 | Very Low Dependency - 6.3% | Contraction | N/A |
| Burkina Faso | 40th percentile - with US$56.8 CHE per capita in 2022 | Moderate Dependency - 20.9% | Expansion | Moderate |
| Burundi | 20th percentile (lower quintile) - with US$24.7 CHE per capita in 2022 | Very High Dependency - 53.2% | Contraction | High |
| Cabo Verde | 80th percentile (Upper quintile) - with US$286 CHE per capita in 2022 | Very Low Dependency - 8.3% | Stagnation | High |
| Cameroon | 40th percentile - with US$72 CHE per capita in 2022 | Low Dependency - 14.4% | Stagnation | High |
| Central African Republic | 20th percentile (lower quintile) - with US$47.9 CHE per capita in 2022 | High Dependency. - 35.6% | Stagnation | High |
| Chad | 20th percentile (lower quintile) - with US$40.2 CHE per capita in 2022 | Moderate Dependency - 18.7% | Expansion | High |
| Comoros | 60th percentile - with US$122.9 CHE per capita in 2022 | High Dependency. - 42.8% | Contraction | High |
| Congo | 20th percentile (lower quintile) - with US$50.6 CHE per capita in 2022 | Moderate Dependency - 15.1% | Contraction | In debt distress |
| Côte d'Ivoire | 60th percentile - with US$86.2 CHE per capita in 2022 | Moderate Dependency - 18% | Expansion | Moderate |
| Democratic Republic of the Congo | 20th percentile (lower quintile) - with US$24.4 CHE per capita in 2022 | High Dependency. - 38.6% | Expansion | Moderate |
| Equatorial Guinea | 80th percentile (Upper quintile) - with US$190.1 CHE per capita in 2022 | Very Low Dependency - 0.8% | Contraction | N/A |
| Eritrea | 20th percentile (lower quintile) - with US$27.1 CHE per capita in 2022 | Moderate Dependency - 22.5% | Stagnation | High |
| Eswatini | 80th percentile (Upper quintile) - with US$283.9 CHE per capita in 2022 | High Dependency. - 28% | Stagnation | N/A |
| Ethiopia | 20th percentile (lower quintile) - with US$27.1 CHE per capita in 2022 | High Dependency. - 25.3% | Stagnation | In debt distress |
| Gabon | 80th percentile (Upper quintile) - with US$247.1 CHE per capita in 2022 | Very Low Dependency - 3.6% | Expansion | N/A |
| Gambia | 20th percentile (lower quintile) - with US$28.9 CHE per capita in 2022 | Moderate Dependency - 24.2% | Stagnation | High |
| Ghana | 40th percentile - with US$82.3 CHE per capita in 2022 | Moderate Dependency - 15.8% | Stagnation | High |
| Guinea | 40th percentile - with US$55 CHE per capita in 2022 | Moderate Dependency - 20.7% | Expansion | Moderate |
| Guinea-Bissau | 40th percentile - with US$66.1 CHE per capita in 2022 | Moderate Dependency - 18.2% | Stagnation | High |
| Kenya | 60th percentile - with US$90.4 CHE per capita in 2022 | Moderate Dependency - 18.4% | Stagnation | High |
| Lesotho | 60th percentile - with US$133.7 CHE per capita in 2022 | High Dependency. - 36.3% | Contraction | Moderate |
| Liberia | 60th percentile - with US$99.7 CHE per capita in 2022 | Moderate Dependency - 19.9% | Contraction | High |
| Madagascar | 20th percentile (lower quintile) - with US$16.3 CHE per capita in 2022 | High Dependency. - 32.8% | Stagnation | Moderate |
| Malawi | 20th percentile (lower quintile) - with US$39.7 CHE per capita in 2022 | Very High Dependency - 64.7% | Stagnation | In distress |
| Mali | 20th percentile (lower quintile) - with US$29.7 CHE per capita in 2022 | Very Low Dependency - 8.7% | Stagnation | Moderate |
| Mauritania | 60th percentile - with US$90.4 CHE per capita in 2022 | Moderate Dependency - 17.5% | Expansion | Moderate |
| Mauritius | 80th percentile (Upper quintile) - with US$590.9 CHE per capita in 2022 | Very Low Dependency - 0.6% | Stagnation | N/A |
| Mozambique | 20th percentile (lower quintile) - with US$49.5 CHE per capita in 2022 | Very High Dependency - 54.2% | Stagnation | High |
| Namibia | 80th percentile (Upper quintile) - with US$405.6 CHE per capita in 2022 | Very Low Dependency - 8.9% | Contraction | N/A |
| Niger | 20th percentile (lower quintile) - with US$26.5 CHE per capita in 2022 | Moderate Dependency - 18.2% | Stagnation | High |
| Nigeria | 60th percentile - with US$90.9 CHE per capita in 2022 | Very Low Dependency - 6.8% | Expansion | N/A |
| Rwanda | 40th percentile - with US$76.8 CHE per capita in 2022 | High Dependency. - 42.9% | Expansion | Moderate |
| Sao Tome and Principe | 80th percentile (Upper quintile) - with US$180.3 CHE per capita in 2022 | High Dependency. - 30.9% | Stagnation | In distress |
| Senegal | 40th percentile - with US$63.7 CHE per capita in 2022 | Moderate Dependency - 21.2% | Expansion | Moderate |
| Seychelles | 80th percentile (Upper quintile) - with US$694.8 CHE per capita in 2022 | Very Low Dependency - 0% | Expansion | N/A |
| Sierra Leone | 20th percentile (lower quintile) - with US$39.3 CHE per capita in 2022 | High Dependency. - 28% | Stagnation | High |
| South Africa | 80th percentile (Upper quintile) - with US$569.8 CHE per capita in 2022 | Very Low Dependency - 1.9% | Contraction | N/A |
| South Sudan | 20th percentile (lower quintile) - with US$49.4 CHE per capita in 2022 | Very High Dependency - 49.1% | Contraction | High |
| Togo | 20th percentile (lower quintile) - with US$54 CHE per capita in 2022 | Moderate Dependency - 21.3% | Expansion | High |
| Uganda | 20th percentile (lower quintile) - with US$44.1 CHE per capita in 2022 | High Dependency. - 41.6% | Expansion | Moderate |
| United Republic of Tanzania | 20th percentile (lower quintile) - with US$35.6 CHE per capita in 2022 | High Dependency. - 38.7% | Expansion | N/A |
| Zambia | 40th percentile - with US$76.1 CHE per capita in 2022 | High Dependency. - 41.4% | Stagnation | In distress |
| Zimbabwe | 40th percentile - with US$70.7 CHE per capita in 2022 | Very High Dependency - 50% | Stagnation | In distress |
