# Supplementary material for: Measuring health financing vulnerability due to reductions in official development assistance: A conceptual framework with empirical application across 47 African countries
Source: PLOS Glob Public Health. 2026 May 6;6(5):e0006282. doi: 10.1371/journal.pgph.0006282 (PMC13148685; doi:10.1371/journal.pgph.0006282)
Supplement: S2 File — (DOCX) [file pgph.0006282.s003.docx]

# **S2 File: Recommendations for countries based on vulnerability category**

All countries can take action to avoid unnecessary deaths and suffering and make progress towards inclusive economic growth, protecting essential health services by prioritizing the most vulnerable populations, protecting their health budgets through increasing efficiency, generating new revenue and earmarking for health through taxes and other sources of funding from global partners, as well as better pooling of resources for health. Some of these actions, depending on the level of vulnerability, require different degrees of fiscal adjustments and health system redesign. The following recommendations are stratified by vulnerability archetypes by country and based on similar topics, but countries are encouraged to adjust as needed, based on their contextual reality.

## ***Countries with very high vulnerability***

- **Data-driven national dialogues and prioritization:** The health sector must engage closely with finance authorities to ensure adequate funding for health programmes, especially those directly impacted by ODA cuts, including social and community insurance where they exist, to build collective solidarity against catastrophic health spending.
- **Pursue health system integration and redesign to ensure cost-efficiency:** Consider cost-efficiency measures in procurement (generics or pooled purchasing) and payroll verification to realize savings that could partly offset lost aid. In the long-term, countries should consider transformative change in health systems design, focusing on an integrated health system approach and avoiding duplication and verticalization, while also accounting for the interlinkages between the different sectors that might be affected by reduction in ODA.
- **Establish immediate fiscal buffers for health: Countries should consider r**ing-fencing sin tax or solidarity-levy revenues for essential health services. In addition, countries should consider creating contingency allocations within the national budget appropriation that can be rapidly redeployed when there are unexpected health sector financial shocks.
- **Negotiate phased donor-transition compacts: Undertake relevant dialogues with donors to c**o-develop 3-to-5-year road maps with major funders, linking aid transition to demonstrable domestic resource mobilization milestones and vulnerability assessment.
- **Mobilize pro-poor concessional financing:** Explore ways and means of securing low-interest or grant-based loans earmarked for essential, high-impact health interventions and engage multilateral development banks and bilateral partners to structure concessional credit lines with extended maturities.
- **Accelerate debt-relief and restructuring talks: Consider p**ursuing common-framework negotiations or IMF programme support to restore debt sustainability and free budget space for health.
- **Institute national coordination and rapid-response expenditure tracking/resource mapping:** Develop a national health resource mapping and convene a regular health sector coordination meeting to update.

## ***Countries with high vulnerability***

- **Data-driven national dialogues and prioritization:** The health sector must engage closely with finance authorities to ensure adequate funding for health programmes, including social and community insurance where these exist, to build collective solidarity against catastrophic health spending.
- **Front-load domestic revenue measures: Consider e**nacting incremental excise increases (for instance, sin taxes like sugar, tobacco or alcohol) or earmarked health levies, in anticipating of donor transitions.
- **Pursue a health system integration and re-design to ensure cost-efficiency audits:** Consider cost-efficiency measures in procurement (generics or pooled purchasing) and payroll verification to realize savings that could partly offset lost aid. In the long-term, countries should consider transformative change in health systems design, focusing on an integrated health system approach and avoiding duplication and verticalization, while also accounting for the interlinkages between the different sectors that might be affected by reduction in ODA.
- **Establish counter cyclical health fund stabilizers: Consider establishing “health stability funds” to d**eposit windfall commodity or tourism revenues into a health stability fund during growth years.
- **Strengthen pooled risk-sharing mechanisms:** Expand national health insurance coverage for the informal sector, with premium subsidies financed through general taxation.
- **Formalize transition dialogues with development partners:** Seek “matching-fund” arrangements, where every additional domestic dollar triggers a proportionate – but declining – donor contribution.

## ***Countries with moderate vulnerability***

- **Data-driven national dialogues and prioritization:** The health sector must engage closely with finance authorities to ensure adequate funding for programmes, ensuring annual health-sector ceilings are commensurately increased based on medium-term revenue forecasts, thereby ensuring gradual yet predictable domestic funding growth.
- **Diversify financing instruments: Consider b**lending concessional loans with performance-based grants, climate-linked bonds, or diaspora bonds earmarked for primary-care infrastructure.
- **Introduce output-based provider payments: Consider** transitioning from input-based line-item budgeting to capitation or diagnosis-related groups to improve value for money, particularly by leveraging social and community insurance where they exist, to build collective solidarity against catastrophic health spending.
- **Public financial management reforms:** Accelerate the roll-out of integrated financial management information systems to cut leakages and improve budget execution rates.
- **Invest in economic-evidence platforms:** Institutionalize health expenditure tracking through national health accounts and fiscal-space analyses to guide domestic health financing decision-making, maintain donor confidence and justify phased aid reallocation.

## ***Countries with low vulnerability***

- **Data-driven national dialogues and prioritization:** The health sector must engage closely with finance authorities to ensure adequate funding for programmes, and also ensure that annual health-sector ceilings are commensurately increased based on medium-term revenue forecasts, and see to gradual yet predictable domestic funding growth.
- **Consider strategic borrowing for health:** Access medium-term loans at concessional rates to finance large-scale health system upgrades (for instance, digital health platforms and hospital expansions), while structuring debt service terms to coincide with projected economic growth and revenue streams, thus minimizing fiscal strain.
- **Increase domestic revenue generation: Consider i**ntroducing or expanding earmarked health levies (for example, sin taxes on tobacco and alcohol) to create predictable funding for priority programmes, while exploring innovative financing instruments (such as health bonds or social impact bonds) to diversify revenue sources.
- **Enhance efficiency and pooling:** Undertake programmatic reviews to eliminate duplicative spending and reallocate savings towards underserved regions or services. Expand pooled procurement mechanisms for medicines and consumables to achieve economies of scale.
- **Develop sustainability plans for external financing:** Co-design multiyear transition plans with donors, outlining tapering schedules and domestic substitution strategies. Align all incoming aid with national budget cycles and health sector priorities, so as to mitigate fiscal cliffs when external support declines.
