# Supplementary material for: Measuring health financing vulnerability due to reductions in official development assistance: A conceptual framework with empirical application across 47 African countries
Source: PLOS Glob Public Health. 2026 May 6;6(5):e0006282. doi: 10.1371/journal.pgph.0006282 (PMC13148685; doi:10.1371/journal.pgph.0006282)
Supplement: Attachment — Submitted filename: Response to Reviewers.pdf [file pgph.0006282.s005.pdf]

3 November 2025

Dear Editor,

Submission of Revised Manuscript - PGPH-D-25-02231

Measuring Health Financing Vulnerability Due to Reductions in Official Development Assistance: A Conceptual Framework with Empirical Application Across 47 African Countries.

We are pleased to submit herewith a revised version of our manuscript entitled ***"Measuring Health Financing Vulnerability Due to Reductions in Official Development Assistance: A Conceptual Framework with Empirical Application Across 47 African Countries."*** for your kind review and consideration for publication in the highly esteemed ***PLOS Global Public Health***.

The authors are extremely grateful to the esteemed reviewers for their critical review, constructive suggestions and feedback which has greatly assisted us to improve the rigor and flow of the manuscript. Attached is a point-by-point response to the comments/feedback received.

We are counting on your continued support and looking forward to hearing from you.

Thank you.

Your sincerely,

Signed by: Dr James Avoka Asamani
Date: 2025-11-04 00:01:13

Prof. James Avoka Asamani
On behalf of the Authors.

RESPONSE TO PEER-REVIEWER'S FEEDBACK

Manuscript number: PGPH-D-25-02231

Evaluation

This paper aims to construct a multidimensional framework to assess the financing vulnerability of health systems in African countries in the context of reductions in external assistance. Through an empirical analysis of 47 African countries, it provides evidence to inform policymaking. The research question is highly relevant and urgent; however, several aspects of the methodology and presentation require further refinement. It is recommended for publication after some revisions.

We thank the reviewer for the kind words, and we are grateful for the constructive feedback.

Comments

Comment 1: The Results section of the Abstract could briefly mention the spatial clustering of highly vulnerable countries, such as those in Eastern and Southern Africa.

Response 1: Thank you for this important suggestion. We have updated the abstract accordingly.

Comment 2: As key background information, Figure 1 should be described in greater detail in the main text. In addition, briefly discussing the underlying reasons for aid cuts would deepen the contextual analysis.

Response 2: Thank you for this suggestion. We have added a paragraph to give additional description on Figure and also added a sentence to ascribe reasons for external aid reductions.

Comment 3: The study adopts the economic vulnerability framework of Briguglio & Galea and Guillaumont. Please clarify why this framework was chosen over others and consider briefly comparing it with existing vulnerability or resilience frameworks to highlight its relative advantages and suitability.

Response 3: We conducted a search for appropriate theoretical and/or analytical frameworks and found that most frameworks focused on the level of vulnerability of households, but not the system-level health financing vulnerability to sudden financial shocks (sudden freeze or withdrawal of funds). The closest framework that could be adapted was the economic vulnerability framework by Briguglio & Galea and Guillaumont. We have revised the first paragraph in page 5 to clarify this.

Comment 4: The analysis covers 47 WHO African Region countries. Please clarify why the other seven African countries were not included, and how their inclusion might have affected the overall findings and regional vulnerability patterns.

Response 4: The analysis was intended to inform WHO's advice and policy response in the African region, which is made up of the 47 countries included in this paper. The other 7 countries in continental Africa belongs to the WHO Eastern Mediterranean

Region and hence were out of the scope of this work. We have now included this in the limitations of the study.

Comment 5: On page 6, when classifying countries into four exposure levels based on current health expenditure per capita, please explain why the 20th, 40th, 60th, and 80th percentiles were chosen. The division and cutoff values from the 60th to the 100th percentile require clarification.

Response 5: In dividing the countries in four groups based on their relative spending, 20th, 40th, 60th and 80th percentiles were chosen because they correspond to the statistical quartiles of the data.

Comment 6: Please check and correct minor editorial issues throughout the manuscript, such as the extra symbol on page 7, line breaks on pages 16 and 19, table numbering on pages 20-21, and the overall clarity of figures.

Response 6: Thank you very much for the observation. We have edited the text and moved the tables and re-numbered them for coherence.

Comment 7: Why are the results of polychoric PCA presented in the Methodology section?

Response 7: Thank you so much for flagging this. We have discussed extensively and reflected on the pros and cons and then took the considered view that the polychoric PCA which only helped us to derive the weights is an essential part of the methods of the paper, but not the main results we seek to disseminate. We considered putting it as a supplementary material, but for completeness of the methodology used, we presented the outcome of the polychoric PCA as part of the method to aid the reader have a fuller understanding of how we came about the weights and then have results section entirely focused on the main thrust of the paper – looking at the vulnerability of countries.

Comment 8: Regarding the "budget space potential" variable, please specify the criteria used by the World Bank to classify countries into "expansion", "stagnation", and "contraction", and provide a corresponding reference.

Response 8: Thank you for this helpful suggestion. We added the following explanation drawn from the World Bank report:

Based on its analysis and projections of real general government expenditure (GGE) per capita, the World Bank classified countries into three categories as either "expansion", "stagnation", or "contraction". According to the analysis, countries in the contraction category are projected to experience absolute declines in real GGE per capita between 2019 and 2029 while countries in the stagnation category is projected to experience sluggish growth in real GGE per capita over the decade from 2019 to 2029. Those in the expansion category are projected to experience substantial growth in real GGE per capita between 2019 and 2029.

Comment 9: Classifying countries not assessed by the IMF/World Bank (N/A) as "low risk" is a reasonable assumption, but this should be explicitly stated, and the potential implications (e.g., possible under- or over-estimation of risk for certain countries) should be discussed.

Response 9: Thank you for pointing out this important issue. We have reflected on it and decided to explicitly acknowledge this point as a limitation of data. We have thus added the following text to the limitation section: "*Furthermore, we assumed that countries not assessed by the IMF/World Bank as "low risk" of debt distress. However, this may not always be the case does not preclude countries in this group having macroeconomic vulnerabilities, which could significantly impact the health financing vulnerabilities derived in the current analysis. Thus, for those countries, under- or over-estimation of the level of health financing vulnerability is a possibility"*

Comment 10: How were the thresholds for vulnerability categories determined (e.g., 40%, 59.5%, and 73% for "low", "high", and "very high" vulnerability)? Please provide a justification or cite relevant methodology.

Response 10: Thank you very much for this suggestion. In the methodology, we have added the following explanation on how the cut-offs were determined: *"These were based on the empirical distribution of vulnerability scores (range 22.6–91.6; mean ≈ 60; standard deviation, SD ≈ 18) which revealed those four natural clusters. Scores ≥ 75% lie about one SD above the mean, defining the upper tail of extreme vulnerability. The 60–73% range approximates values around the mean ± 0.5 SD, representing moderately elevated vulnerability. Scores between 40–59.5% fall roughly one SD below the mean, indicating moderate vulnerability, while scores < 40% occupy the lower quartile."*

Comment 11: In Figure 8, why do the coefficients of the two equations differ? Including two different equations in the same figure is puzzled.

Response 11: Thank you very much for meticulously picking this up. We have carefully reviewed Figure 8. One of the equations was a redundant text from other sensitivity analysis we were exploring. We have deleted the redundant text.

Comment 12: The slight decrease in explanatory power (R^2 dropped from 31% to 28%) when the poverty variable was included in Table 5 requires discussion.

Response 12: We now integrated an extensive discussion around the role of poverty as follows:

"Despite its conceptual relevance, we observe that slight decrease in explanatory power (R^2 dropped from 31% to 28%) when the poverty variable was included. This may not necessarily suggest that poverty was a less important variable but may require further assessment to understand the nature of its contribution. One key challenge we observed is that the poverty data for most countries are outdated (in some cases about 5 to 10 years old) as it is derived from national surveys which are not always regular or the same year for all countries. As a result, temporality with the rest of the variables included in the analysis appear to be limited."

Comment 13: The Discussion could benefit from a more in-depth case-style analysis of representative countries to illustrate why they fall into specific vulnerability categories.

Response 13: In the discussion, we already contrasted Malawi, Nigeria and South Africa, but based on this comment, we have added a box to discuss Malawi and Nigeria as a case study, representing the extremes of vulnerability categories.

Comment 14: Policy recommendations could be more specific and tailored to countries with “high”, “moderate”, and “low” levels of vulnerability, respectively.

Response 14: Due to the space constraints, we have added a supplementary material in which we have tailored the recommendations based on the countries’ level of vulnerability.

Comment 15: The dynamic aspect of the framework could be further emphasized. It may be beneficial to periodically update the vulnerability scores to reflect policy changes, and a regular reassessment mechanism might be considered.

Response 15: Thank you for this important suggestion. We have now included this in the recommendations.
