# Supplementary material for: Measuring health financing vulnerability due to reductions in official development assistance: A conceptual framework with empirical application across 47 African countries
Source: PLOS Glob Public Health. 2026 May 6;6(5):e0006282. doi: 10.1371/journal.pgph.0006282 (PMC13148685; doi:10.1371/journal.pgph.0006282)
Supplement: Attachment — Submitted filename: Point-by-Point Response to Peer-Reviewers_PGPH-D-25-02231.pdf [file pgph.0006282.s006.pdf]

29 November 2025

Dear Editor,

Submission of Second Revision of Manuscript - PGPH-D-25-02231

Measuring Health Financing Vulnerability Due to Reductions in Official Development Assistance: A Conceptual Framework with Empirical Application Across 47 African Countries.

We are pleased to submit herewith a second revision of our manuscript entitled ***"Measuring Health Financing Vulnerability Due to Reductions in Official Development Assistance: A Conceptual Framework with Empirical Application Across 47 African Countries."*** for your kind review and consideration for publication in the highly esteemed ***PLOS Global Public Health***.

The authors are extremely grateful to the esteemed reviewers for their critical review, constructive suggestions and feedback which has greatly assisted us to improve the rigor and flow of the manuscript. Attached is a point-by-point response to the comments/feedback received.

We are counting on your continued support and looking forward to hearing from you.

Thank you.

Your sincerely,

Prof. James Avoka Asamani

On behalf of the Authors.

RESPONSE TO PEER-REVIEWER'S FEEDBACK

Manuscript number: PGPH-D-25-02231

Comment 1: The authors have adequately addressed most of my previous concerns. The manuscript has been significantly improved through revisions that enhance its clarity, methodological rigor, and policy relevance.

Response 1: We thank the esteemed reviewer for the kind thorough review and constructive feedback as well as the kind words. We are encouraged.

Comment 1: The authors' response that the 20th, 40th, 60th, and 80th percentiles correspond to quartiles is statistically inaccurate (quartiles are the 25th, 50th, and 75th percentiles). The use of the 20th, 40th, 60th, and 80th percentiles effectively divides the data into five groups, which contradicts the stated goal of "classifying countries into four exposure levels." Please provide a clear and correct justification for the chosen grouping method.

Response 2: We thank the esteemed reviewer for the careful and insightful observation. We fully acknowledge the inaccuracy in our earlier response regarding the correspondence between the 20th, 40th, 60th, and 80th percentiles and statistical quartiles.

The choice of the 20th, 40th, 60th, and 80th percentiles as explained the manuscript, was chosen to enable us to capture finer gradations in countries' per capita health expenditure levels than would have been possible with conventional quartiles. Several countries with meaningfully different vulnerability profiles would have been grouped together if we had used quartiles, thereby masking important heterogeneity in vulnerability. Using quintile-like cut points enabled us to better differentiate these nuanced variations while still maintaining an analytically coherent classification structure.

We have now clarified this justification in the manuscript to avoid any ambiguity.

Comment 1: Furthermore, should other datasets beyond the poverty data also suffer from timeliness issues, the discussion section should be expanded to address the implications of these limitations for the proposed framework.

Response 3: We appreciate the reviewer's valuable observation. We have clarified in the discussion section that the replicability and policy-tracking value of the proposed measurement framework depend on the routine and timely updating of all underlying datasets. The sensitivity observed with the poverty data in this analysis illustrates that delays or irregular updates in any of the input variables can affect the accuracy and responsiveness of the framework.

We have further elaborated on these implications in the revised discussion.

Comment 4: The authors are encouraged to incorporate relevant recent literature, such as the

following studies in China, to further strengthen the study's justification and discussion:
doi: 10.1016/S2468-2667(22)00256-0
doi: 10.1016/j.glohj.2021.08.001
doi: 10.1186/s12939-019-0982-6

Response 4: Thank you for sharing these useful papers from China. We have reviewed these papers and incorporated in some of the discussion as appropriate.
