# Supplementary material for: Measuring health financing vulnerability due to reductions in official development assistance: A conceptual framework with empirical application across 47 African countries
Source: PLOS Glob Public Health. 2026 May 6;6(5):e0006282. doi: 10.1371/journal.pgph.0006282 (PMC13148685; doi:10.1371/journal.pgph.0006282)
Supplement: Attachment — Submitted filename: Comments to the Author-PGPH-D-25-02231.docx [file pgph.0006282.s004.docx]

***Evaluation***

This paper aims to construct a multidimensional framework to assess the financing vulnerability of health systems in African countries in the context of reductions in external assistance. Through an empirical analysis of 47 African countries, it provides evidence to inform policy-making. The research question is highly relevant and urgent; however, several aspects of the methodology and presentation require further refinement. It is recommended for publication after some revisions.

***Comments***

1. The Results section of the Abstract could briefly mention the spatial clustering of highly vulnerable countries, such as those in Eastern and Southern Africa.
2. As key background information, Figure 1 should be described in greater detail in the main text. In addition, briefly discussing the underlying reasons for aid cuts would deepen the contextual analysis.
3. The study adopts the economic vulnerability framework of Briguglio & Galea and Guillaumont. Please clarify why this framework was chosen over others, and consider briefly comparing it with existing vulnerability or resilience frameworks to highlight its relative advantages and suitability.
4. The analysis covers 47 WHO African Region countries. Please clarify why the other seven African countries were not included, and how their inclusion might have affected the overall findings and regional vulnerability patterns.
5. On page 6, when classifying countries into four exposure levels based on current health expenditure per capita, please explain why the 20th, 40th, 60th, and 80th percentiles were chosen. The division and cutoff values from the 60th to the 100th percentile require clarification.
6. Please check and correct minor editorial issues throughout the manuscript, such as the extra symbol on page 7, line breaks on pages 16 and 19, table numbering on pages 20-21, and the overall clarity of figures.
7. Why are the results of polychoric PCA presented in the Methodology section?
8. Regarding the “budget space potential” variable, please specify the criteria used by the World Bank to classify countries into “expansion”, “stagnation”, and “contraction”, and provide a corresponding reference.
9. Classifying countries not assessed by the IMF/World Bank (N/A) as “low risk” is a reasonable assumption, but this should be explicitly stated, and the potential implications (e.g., possible under- or over-estimation of risk for certain countries) should be discussed.
10. How were the thresholds for vulnerability categories determined (e.g., 40%, 59.5%, and 73% for “low”, “high”, and “very high” vulnerability)? Please provide a justification or cite relevant methodology.
11. In Figure 8, why do the coefficients of the two equations differ? Including two different equations in the same figure is puzzled.
12. The slight decrease in explanatory power (R^2^ dropped from 31% to 28%) when the poverty variable was included in Table 5 requires discussion.
13. The Discussion could benefit from a more in-depth case-style analysis of representative countries to illustrate why they fall into specific vulnerability categories.
14. Policy recommendations could be more specific and tailored to countries with “high”, “moderate”, and “low” levels of vulnerability, respectively.
15. The dynamic aspect of the framework could be further emphasized. It may be beneficial to periodically update the vulnerability scores to reflect policy changes, and a regular reassessment mechanism might be considered.
